# Respiratory DC Use IFITM3 to Avoid Direct Viral Infection and Safeguard Virus-Specific CD8^+^ T Cell Priming

**DOI:** 10.1371/journal.pone.0143539

**Published:** 2015-11-23

**Authors:** Giuseppe Infusini, Jeffrey M. Smith, He Yuan, Angela Pizzolla, Wy Ching Ng, Sarah L. Londrigan, Ashraful Haque, Patrick C. Reading, Jose A. Villadangos, Linda M. Wakim

**Affiliations:** 1 Division of Systems Biology and Personalised Medicine, The Walter and Eliza Hall Institute of Medical Research, Parkville, VIC 3052, Australia; 2 Department of Microbiology and Immunology, The University of Melbourne, at the Peter Doherty Institute for Infection and Immunity, Melbourne, Victoria 3000, Australia; 3 Malaria Immunology Lab, QIMR Berghofer Medical Research Institute, Brisbane, Australia; 4 WHO Collaborating Centre for Reference and Research on Influenza, Victorian Infectious Diseases Reference Laboratory, at the Peter Doherty Institute for Infection and Immunity, Melbourne, Victoria 3000, Australia; 5 Department of Biochemistry and Molecular Biology, Bio21 Molecular Science and Biotechnology Institute, The University of Melbourne, Parkville, VIC 3010, Australia; Imperial College London, UNITED KINGDOM

## Abstract

Respiratory dendritic cells (DC) play a pivotal role in the initiation of adaptive immune responses to influenza virus. To do this, respiratory DCs must ferry viral antigen from the lung to the draining lymph node without becoming infected and perishing en route. We show that respiratory DCs up-regulate the expression of the antiviral molecule, interferon-induced transmembrane protein 3 (IFITM3) in response to influenza virus infection, in a manner dependent on type I interferon signaling and the transcription factors IRF7 and IRF3. Failure of respiratory DCs to up-regulate IFITM3 following influenza virus infection resulted in impaired trafficking to the draining LN and consequently in impaired priming of an influenza-specific CD8+ T cell response. The impaired trafficking of IFITM3-deficient DC correlated with an increased susceptibility of these DC to influenza virus infection. This work shows that the expression of IFITM3 protects respiratory DCs from influenza virus infection, permitting migration from lung to LN and optimal priming of a virus specific T-cell response.

## Introduction

Type 1 interferons (IFN) are components of the innate immune response released as a first line of defense upon encounter of viruses and other intracellular pathogens. They induce the transcription of IFN-stimulated (or–regulated) genes (ISGs) which encode factors that act “extrinsically” by recruiting other molecular and cellular components of the innate and adaptive immune response, and “intrinsically” by antagonizing viral replication in infected cells [1]. One family of ISGs that act via intrinsic mechanisms are the interferon-induced transmembrane (IFITMs) proteins. The IFITMs restrict infection of a diverse range of viruses [2]. IFITM3 is a member of this family that is particularly effective at controlling influenza A virus (IAV) infection. Mice lacking IFITM3 are highly susceptible to IAV infection even when challenged with a normally low-pathogenic strain, and humans expressing a functionally defective IFITM3 allelic variant are likewise more susceptible to IAV [3] [4]. IFITM3 expression can be induced in all cells, so defective IFITM3 function may lead to an increased susceptibility of most, if not all cell types to IAV infection and this in turn may contribute to enhanced pathogenicity in mice or humans. However, there is an alternative possibility, namely that the cells that need to be protected foremost are the components of the immune system involved in fighting the virus and preventing re-infection. This is plausible because the immune response against IAV infection is highly protective and essential for successful control of the virus. Furthermore, we have previously described an important role for IFITM3 in prophylactic protection of tissue resident memory CD8 T cells, a population of lymphocytes that remain at sites of infection following the induction of a primary immune response and clearance of the pathogen [5]. By maintaining expression of IFITM3, IAV-specific tissue resident memory T cells contained in the lung mucosa can withstand viral infection during a secondary challenge and effect quick protection at the site of viral entry. Mice in which these cells lack IFITM3 are more susceptible to secondary IAV infection. These studies helped us establish the critical role played by the relatively small number of tissue resident memory CD8 T cells in protection against secondary IAV infection [5].

Another type of cell of the immune system that is obligatorily involved in induction of protective CD8 T immunity against IAV are professional antigen presenting cells. The lung mucosa contains three major types of such cells: macrophages, CD11b+ DC and CD103+ DC [6] [7]. It is well established that DC play the predominant role in transporting and presenting viral antigen via MHC I (either through the classical or the cross-presentation pathway) to CD8 T cells in the mediastinal lymph node (LN) where naïve T cells are primed against lung infections [8] [9]. Furthermore, for a full anti-viral response to occur, and for generation and re-stimulation of memory CD8 T cell responses, antigen presentation is required at both the LN and at the site of infection itself [10], and different DC may be involved at either location. Regardless of the specific function that distinct DC types may play in immunity against IAV, a mechanism to protect these cells against the deleterious effects of viral infection would be beneficial, but no such mechanism has been described yet.

Here we show respiratory DCs up-regulate expression of IFITM3 in response to IAV infection. Expression of IFITM3 prevented death of respiratory DCs upon IAV infection *in vivo*, permitting their migration from lung to LN to effect optimal antigen presentation and priming of an IAV-specific T-cell response. Our results extend the range of functions of IFITM3 in the immune system and help in understanding the function of respiratory DC in the induction of immune responses against IAV infection.

## Results

### IAV-induced up-regulation of ifitm3 on mouse respiratory antigen presenting cells

**Fig 1:**
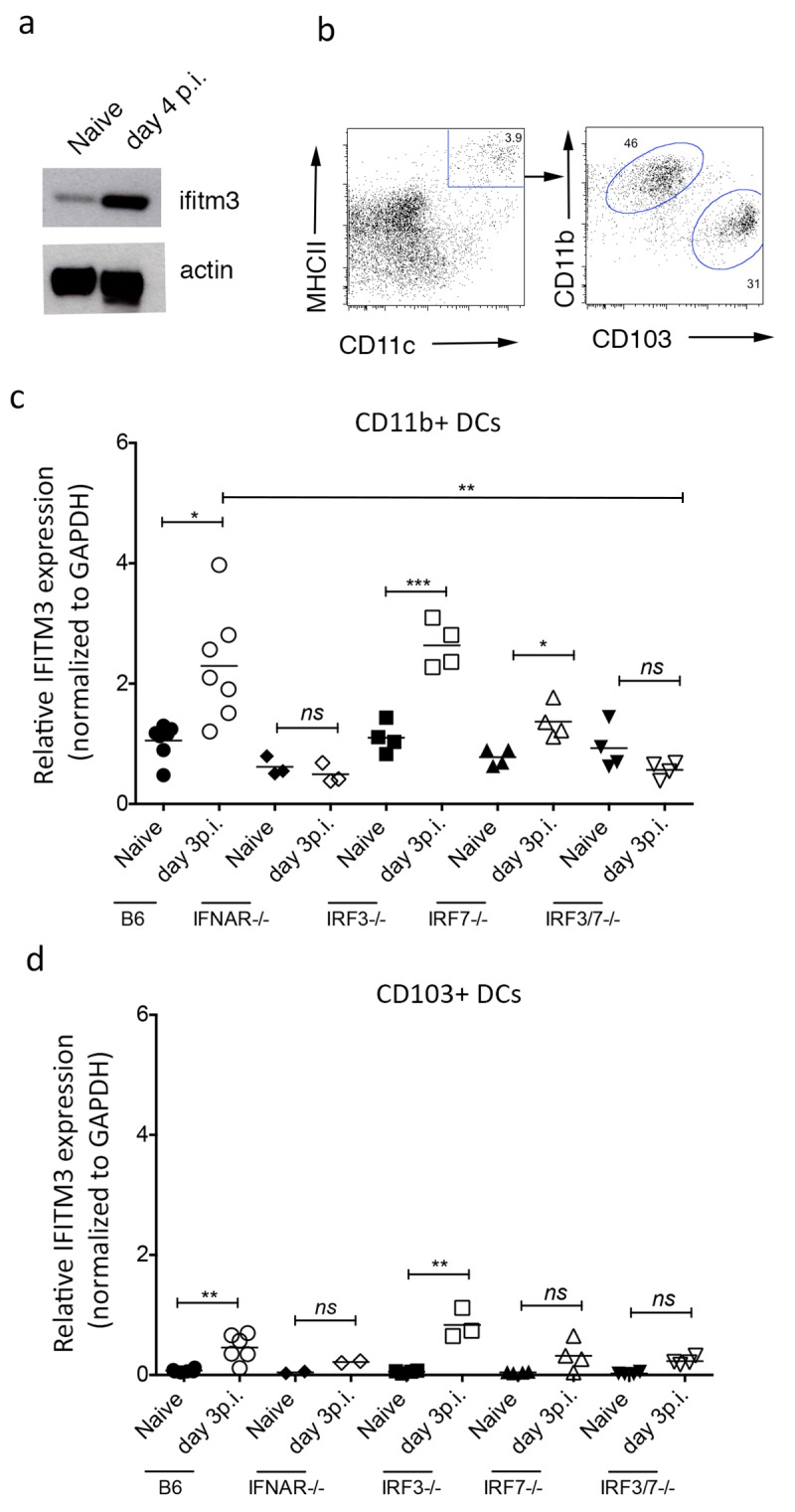
Influenza virus induced up-regulation of IFITM3 on respiratory APCs is dependent on type I interferon signaling. (a) The expression of IFITM3 protein in bulk DCs sort purified from the lung of mice either before (naïve) or 4 days after intranasal infection with IAV. (b) Representative flow cytometry profiles depicting the two DCs populations in the mouse lung. The expression of IFITM3 mRNA measured by real time PCR in (c) CD11b+ DCs and (d) CD103+ DCs sort purified from the lungs of B6, IFNAR-/-, IRF3 KO, IRF7 KO or IRF3/7 KO mice that were naïve or had been infected 3 days previously with IAV. The expression of IFITM3 mRNA in each subset was measured by real time PCR. Values normalized to the housekeeping gene GAPDH. (n = 2–3 mice pooled per experiment, data pooled from 4 independent experiments, Student’s t-test, *P <0.05, **P<0.01, ***P<0.001, *ns* = *P*>0.05).

To determine whether IFITM3 plays a role in protecting mouse lung antigen presenting cells (APCs) during IAV infection, we first assessed the expression of IFITM3 protein in bulk DCs (CD11+MHC +) isolated from the lungs of mice either before or 4 days after intranasal infection with IAV (x31 strain). We detect low levels of IFITM3 protein in DCs isolated from the lungs of naïve mice and this expression increased following influenza infection (**Fig 1A**). We next measured the *ifitm3* expression in the two major respiratory DC populations, CD11b+ and CD103+ DCs (**Fig 1B**), before or 3 days after intranasal infection with IAV (x31 strain). Both subsets showed increased expression of *ifitm3* (2–4 fold) following infection (**Fig 1C and 1D)**. The stimulus that induced *ifitm3* was type I interferon because APCs lacking the type I interferon receptor (IFNAR-/-) did not up-regulate expression (**Fig 1C and 1D)**.

The transcription factors IRF-3 and IRF-7 are master regulators of type I IFN-stimulated gene expression [11]. To determine which of these transcription factors was involved in up-regulation of *ifitm3* expression in mice infected with IAV we isolated respiratory APCs from naïve or IAV-infected IRF3-, IRF7- and IRF3/7-deficient mice. Both respiratory antigen presenting cells lacking IRF3 or IRF7 elevated *ifitm3* expression following IAV infection, but cells lacking IRF7 together with IRF3 did not (**Fig 1C and 1D**). The amount of ifitm3 transcript on day 3 post infection detected in CD11b+ DCs lacking IRF3 and IRF7 was significantly lower than the levels expressed in B6 controls. These data indicate that antigen presenting cells within the lung increase expression of IFITM3 following influenza virus infection, in a manner dependent on type I interferon signaling and the transcription factors IRF7 and IRF3.

### IFITM3-deficient DCs are more susceptible to influenza virus infection

**Fig 2:**
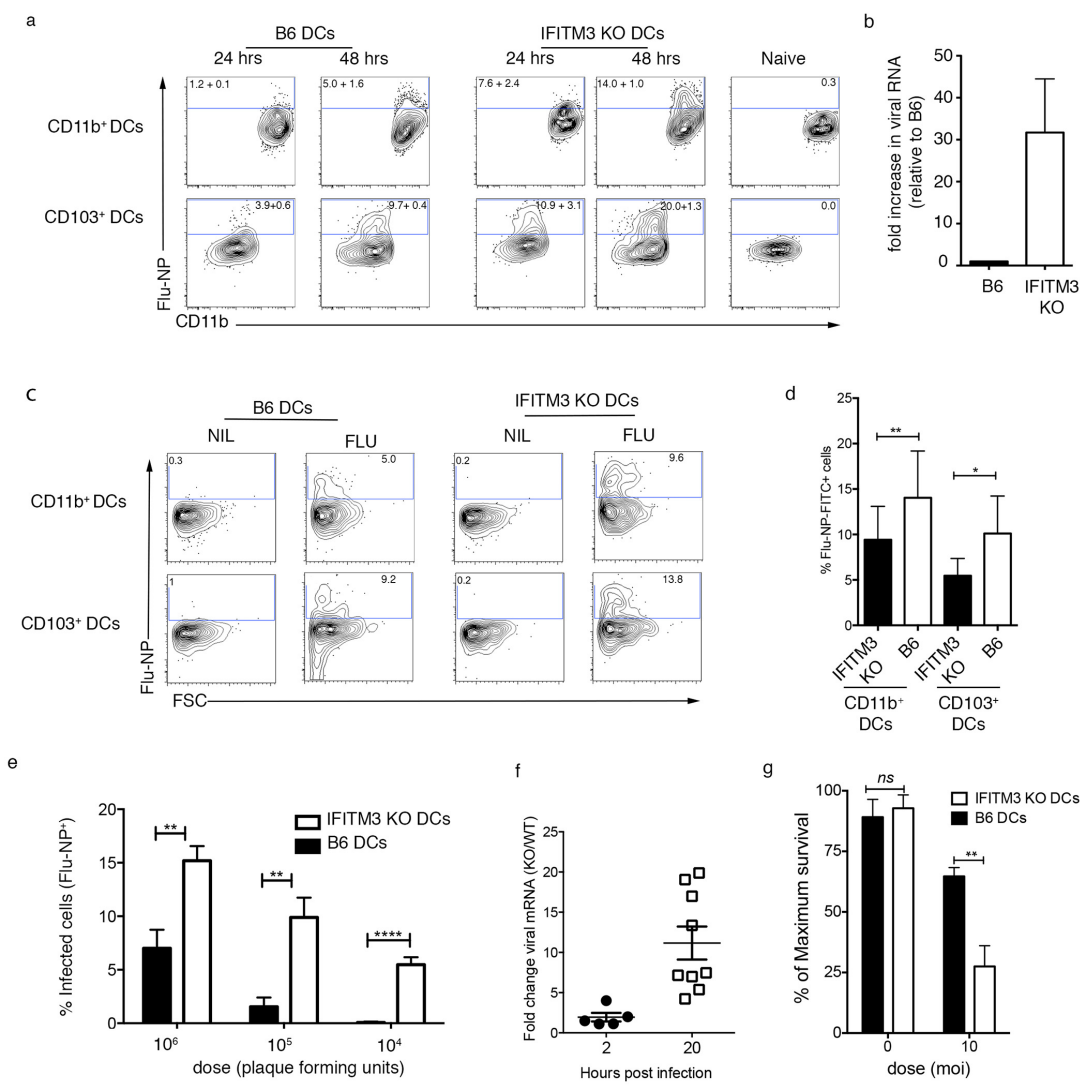
IFITM3 KO DCs are more susceptible to influenza virus infection. (a) B6 or IFITM3 KO mice were infected via the intranasal route with 10^4^ PFU of influenza virus (Pr8) and 24 or 48 hrs later mice were culled and single cell preparations of lung cells were stained for expression of viral NP. The proportion of lung DCs staining positive for influenza virus NP was then determined by flow cytometry. Representative flow cytometry plots are shown (n = 4–6 mice, data pooled from 3 independent experiments, numbers represent mean + sem) (b) Dendritic cells (CD11c+MHCII+ F4/80-) were sort purified from the lungs of with B6 or IFITM3 KO mice 48 hrs after intranasal infection with 10^4^ PFU of influenza virus (Pr8). The total amount of viral RNA present in the DC population was measured by real time PCR. Data are pooled from 3 independent experiments. Graph represents the fold increase in viral RNA normalized to the amount of viral RNA present in the B6 DCs. (c-d) CD103+ and CD11b+ DC were sort purified from the lungs of B6 or IFITM3 KO mice infected with influenza virus (moi 10) and cells were fixed 24 hr later, stained for influenza virus NP and examined by flow cytometry. (c) Representative flow cytometry profiles are shown. Data is representative of 3 independent experiments. (d) Graph depicts the mean percent infection + sem (data is pooled from 3 independent experiments, Student’s t test, **P<0.01, ***P<0.001) (e) DCs were sort purified from the spleens of B6 and IFITM3 KO mice and monolayers of cells were infected with influenza virus at the doses indicated. At 12 hr post-infection, cells were fixed and stained for expression of viral NP and the percentage of infected cells was determined in at least 5 random fields (>200 cells). Data show the mean percent infection + sem (data is pooled from 3 independent experiments, Student’s t test, **P<0.01, ***P<0.001, ****P<0.0001) (f) Bone marrow derived B6 or IFITM3 KO DCs were infected with IAV (moi 1) and 2 and 20 hours later the amount of viral mRNA was measured by qRT-PCR. Graph represents the fold increase in viral RNA in IFITM3 KO DCs relative to the amount present in the B6 DCs (data pooled form 5–9 experiment, symbols represent individual experiments) (g) DCs purified from the spleens of B6 and IFITM3 KO mice were infected with IAV (moi 10) or were left uninfected and 12 hours later the proportion of viable cells was measured by flow cytometry. Graphs depict the percentage of maximum survival (data pooled from 2 independent experiments, Student’s t test, **P<0.01).

We next examined whether DC are indeed protected from IAV infection by the cell-intrinsic anti-viral properties of IFITM3. We firstly assessed the susceptibility of respiratory DCs to IAV infection in vivo by determining the number of respiratory DCs isolated from wild-type B6 and IFITM3-deficient mice that stain positive for influenza virus nuclear protein (NP) 24 or 48 hrs after intranasal influenza virus infection. We observed a ~2–6 fold increase in the proportion of NP+ DCs in the absence of IFITM3 (**Fig 2A**). Furthermore, IFITM3-deficient DC isolated from the lungs of mice 48 hrs after IAV infection harbored ~30 times more viral RNA than DC from control wild-type B6 mice (**Fig 2B**). We next assessed the viral susceptibility of respiratory DCs *in vitro*, to ensure that the infectability of wild type and IFITM3 deficient DCs was assessed in the presence of equal amounts of virus. To do this we sort purified CD11b+ and CD103+ DCs from the lungs of naïve B6 or IFITM3 deficient mice and infected these cells in vitro with IAV. Flow cytometry was then used to detect expression of viral NP 24 hrs later. We observed a ~1.5-2-fold increase in the proportion of NP+ DCs in the absence of IFITM3 (**Fig 2C and 2D**). Splenic conventional DCs lacking IFITM3 also displayed enhanced susceptibility to influenza virus infection. To show this we purified conventional DC from the spleens of B6 and IFITM3 KO mice, infected them with varying doses of IAV and determined the percentage of infected cells 12 hrs later by measuring the number of cells staining positive for IAV nuclear protein (NP). IFITM3 KO DC were more susceptible to infection compared to control cells at all the tested virus doses (**Fig 2E**), but especially when a low virus inoculum was used. We also measured infectability of IFITM3 KO DCs by quantitating the amount of IAV mRNA, an intermediate only synthesized during virus replication. To do this bone marrow derived B6 or IFITM3 KO DCs were infected with IAV and at 2 and 20 hrs post infection the amount of viral mRNA was measured by qRT-PCR. We observed a 2–10 fold increase in the amount viral mRNA in IFITM3 KO DCs compared to the B6 controls (**Fig 2F**). We next determined if the increase in NP+ cells and viral mRNA observed in IFITM3 KO DCs correlated with an increase in cell death. To assess this, we purified conventional DC from the spleens of B6 and IFITM3 KO mice, infected them with IAV (moi 10) and determined the percentage survival 12 hrs later. We observe a significant increase in cell death of IFITM3 KO DCs compared to B6 DCs controls following 12 hrs of exposure of influenza virus (**Fig 2G**). Together, these data show that IFITM3 expression increases the resistance of DC to IAV infection, consistent with its function in other cell types.

### Delayed induction of an influenza specific CD8+ T cell response in IFITM3 KO mice

**Fig 3:**
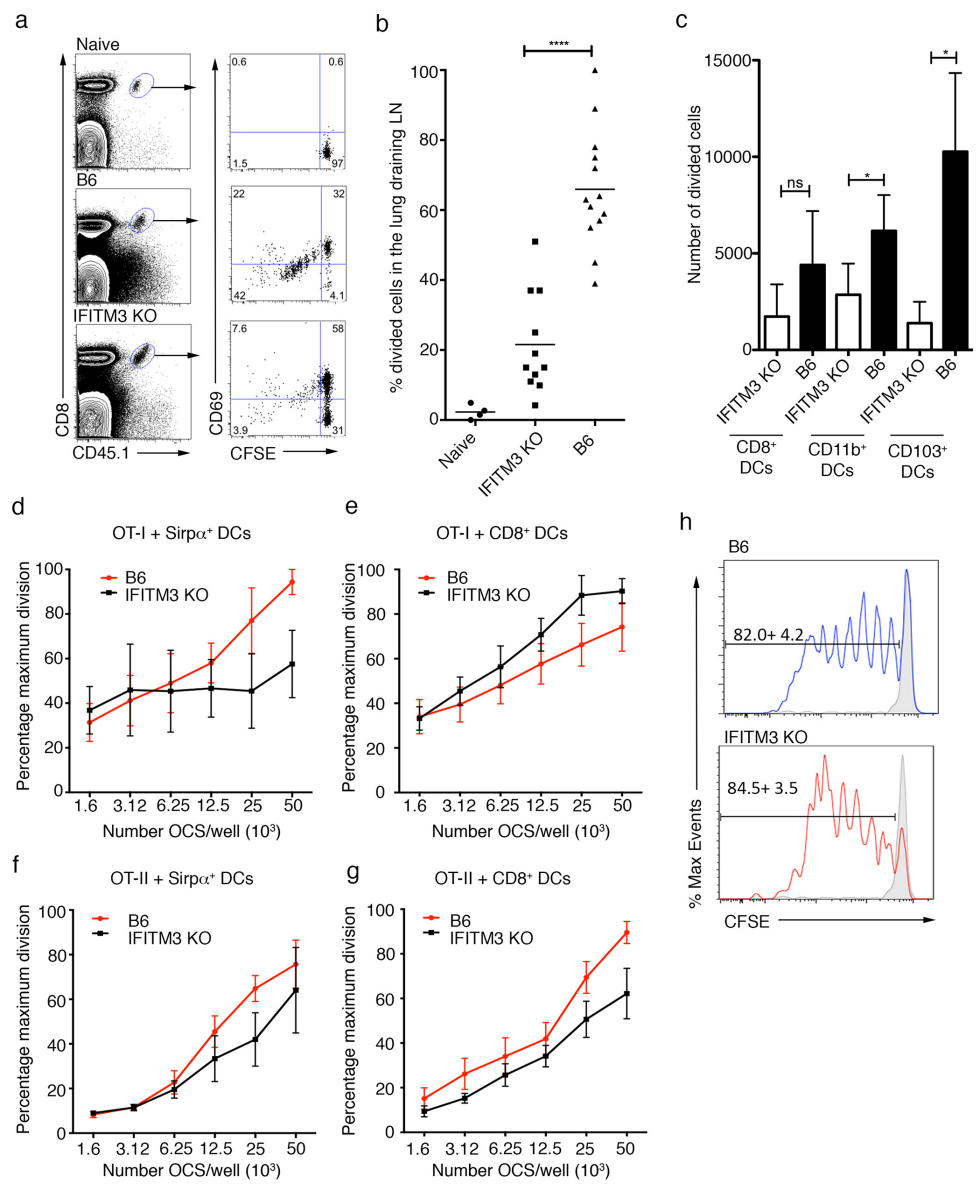
Delayed CD8+ T cell activation in IFITM3 KO mice following influenza virus infection. B6 (wild type) or IFITM3 KO mice were seeded with naïve CFSE-labeled OT-I.CD45.1 one day prior intranasal infection with with 10^4^ PFU of Flu-OVA (x31-OVA). The percentage of divided cells in the lung-draining LN was subsequently measured 72hrs later (a) Representative flow cytometry profiles gated on OT-I.CD45.1 (CD8+CD45.1+) cells in the lung-draining LN depicting CFSE dilution and CD69 up-regulation. (b) The percentage of divided cells in the LN is shown. Dots represent individual mice, bars represent the mean. (*n* = 4–13, data pooled from 4 independent experiments, Student’s t-test, ****P<0.0001) (c) CD8+, CD103+ and CD11b+ DCs were sort purified from the lung-draining LN of mice 2 days following i.n. infection with 10^4^ PFU of Flu-OVA (x31-OVA) and were cultured with CFSE labeled OT-I cells. After 3 days, proliferation of OT-I T cells was analyzed by flow cytometry. The absolute number of divided cells is shown. Bars represent mean ± sem. (n = 5 mice pooled per experiment, data pooled from three independent experiments, Student’s t-test, *P <0.05, *ns* = *P*>0.05). (d-g) Sirpα+ and CD8+ splenic DCs were sort purified from the spleens of either wild type (B6) of IFITM3 KO mice and cultured with serial dilutions of OVA-coated splenocytes and CFSE-labeled OT-I (d-e) or OT-II (f-g) cells. After 3 days, proliferation of OT-I and OT-II T cells was analyzed by flow cytometry. Graphs show the relative proliferation seen as a percentage of the maximum proliferation per assay. Data are pooled from 3 independent experiments and represent percentage maximum division ± SEM. (h) B6 or IFITM3 KO mice were seeded with naïve CFSE-labeled OT-I.CD45.1 one day prior to intranasal injection of OCS (open histograms) or saline (grey histograms). The percentage of divided cells in the lung-draining LN was measured 72hrs later. Representative flow cytometry profiles are depicted, mean division + sem is shown (n = 5, data pooled from 2 experiments).

We next determined if the expression of IFITM3 by respiratory DC was essential for these cells to prime an anti-IAV CD8+ T cell response. To do this, wild-type and IFITM3 KO mice were seeded with CFSE-labeled naïve OVA-specific OT-I.CD45.1 TCR transgenic CD8+ T cells prior to intranasal infection with an influenza virus engineered to express the model antigen ovalbumin (Flu-OVA). We assessed priming 72 hrs later by measuring proliferation and up-regulation of the activation marker CD69 on OT-I T cells isolated from the lung-draining LN. While ~60% of OT-I T cells had undergone division in wild-type mice, only ~20% had divided in IFITM3-deficient animals (**Fig 3A and 3B**).

An impairment in antigen presentation by IFITM3-deficient DCs was also evident when DC subsets were purified from the lung-draining LN of wild-type or IFITM3-deficient mice 48 hrs after Flu-OVA infection and cultured *in vitro* with CFSE labeled naïve OT-I cells. We examined the two DC subsets that immigrate into the LN from lung tissue, designated CD11b+ and CD103+ DC and the LN resident CD8+ DC. Assessment of the expression of activation markers, CD80 and CD86 revealed equivalent expression on both B6 and IFITM3 KO DCs (**S1 Fig**). As described by others, all three populations could present virally derived antigen to some extent [9] [12] [13] [8], though the migratory CD103+ DC were the more efficient on a per-cell basis *ex vivo* [8]. Both migratory DC subsets lacking IFITM3 had impaired capacity to present antigen to OT-I T cells (**Fig 3C**). The impairment might be due to defective processing and/or presentation of virally derived antigens, or to deficient migration of DC carrying virally derived antigen from the viral infected lung to the draining LN, so these two possibilities were investigated as potential mechanisms affected by IFITM3 expression.

### IFITM3 KO DCs display normal antigen presentation ability

We assessed the ability of IFITM3-deficient DCs to present antigen in vitro. We purified the two main subsets of conventional DC, namely CD8+ DCs and Sirpa+ DCs, from the spleens of B6 or IFITM3-deficient mice and cultured them with OVA-coated splenocytes and CFSE-labeled OT-I or OT-II cells to assess class I and class II antigen (cross)presentation, respectively. The number of T cells undergoing division was measured 60 hrs later. Control DC and DC deficient in IFITM3 could process and present OVA with similar efficiency in this assay (**Fig 3D–3G**). We also assessed presentation of non-infectious antigen *in vivo*. Wild-type and IFITM3-deficient mice were seeded with naïve CFSE-labeled OT-I cells and immunized with OVA-coated splenocytes by the intranasal route. OT-I division was measured 72 hours later in the lung draining LN (**Fig 3H**). We observed equivalent levels of OT-I division in both groups of mice indicating that DC lacking IFITM3 are capable of effective cross presentation of inert antigen *in vivo*. Overall, the data implies that the observed impairment in CD8 T cell priming in mice deficient in IFITM3 following IAV infection is unlikely due to an intrinsic impairment in antigen presentation function in DC.

### Lack of IFITM3 impairs DC migration from lung mucosa to lymph nodes upon IAV infection

An alternative explanation for the impaired priming of IAV-specific CD8 T cells in IFITM3 deficient mice is that the DC from the deficient mice do not traffic as efficiently to the draining LN as DCs from wild-type mice. If this were the case, these DC would not be capable of efficiently presenting the antigen in the LN themselves, nor would they transfer antigen captured in the lung mucosa to LN-resident DC, two of the major activities associated with priming CD8+ T cell responses that have been attributed to migratory DC [8, 9]. To assess this possibility, we instilled IAV and fluorescent beads intranasally into wild type or IFITM3-deficient mice. We examined the localization and measured the number of DC bearing beads in the LN 48hrs later.

**Fig 4:**
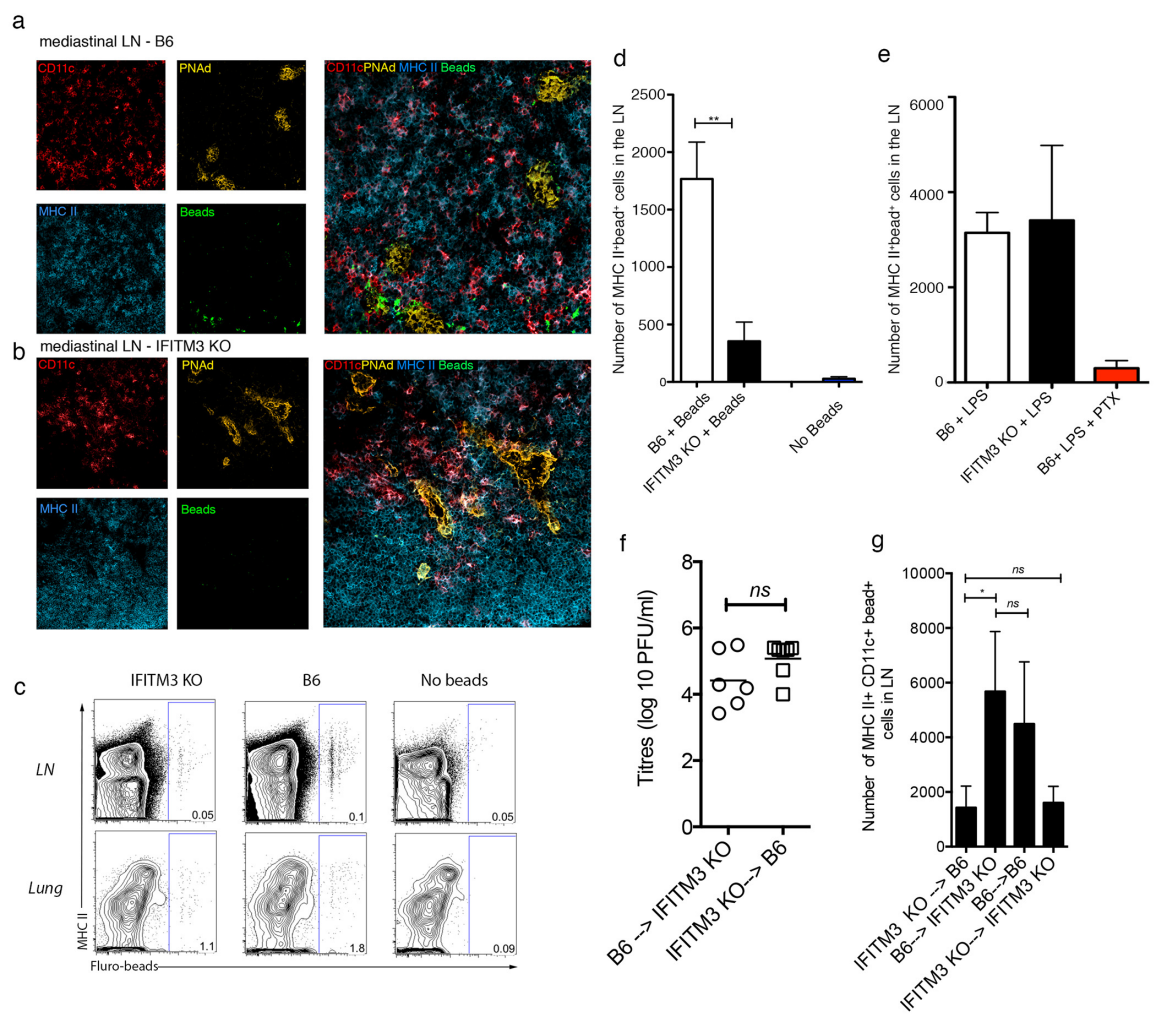
IFITM3 KO DCs are impaired in trafficking to the draining LN following influenza virus infection. B6 or IFITM3 KO mice were intranasally instilled with fluorescent-beads and influenza virus. Lungs and lung-draining LN were harvested 48hrs later. Immunofluorescent staining of LN sections from (a) B6 and (b) IFITM3 KO mice at 48hrs post-infection. Staining for CD11c (red), MHC II (blue), PNad (yellow) and beads (green). x20 magnification, representative for 2 independent experiments (c) Representative flow cytometry profiles depicting beads within MHC II+ DCs in lung and LN. (d) The absolute number of bead+ MHC-II+ DCs in the draining LN (*n* = 5 mice per group, data pooled from 2 independent experiments, Student’s t-test **P <0.01) (e) B6 and IFITM3 KO mice were intranasally instilled with fluorescent-beads and LPS +/- pertussis toxin (PTX). The absolute number of bead+ MHCII+ DCs in the lung-draining LN was determined 48hrs later. Bars represent the mean + sem (*n* = 5, data pooled from 2 independent experiments) (f-g) B6—>IFITM3 KO, IFITM3 KO→B6, B6→B6 and IFITM3 KO→IFITM3 KO mice were intranasally instilled with fluorescent-beads and influenza virus. (f) Viral titres in the lung on day 2 post infection was measured by standard plaque forming unit assay (g) Lungs draining LN were harvested 48hrs later and the absolute number of bead+ MHC-II+ DCs was determined (*n* = 5–8 mice per group, data pooled from 3 independent experiments, Student’s t-test **P <0.01).

Images shown in Fig 4A and 4B show localization of beads in CD11c+MHCII+ cells congregated around high endothelia vessels in the LN of wild type mice. Bead+ cells were much more scarce in the LN of IFITM3-deficient mice. In additional experiments, single cell suspensions prepared from LN and lung were analyzed for the presence of fluorescent beads using flow cytometery. Of interest, we observed a 4-fold reduction in the number of bead+ MHC II+ CD11c+ DC in the LN of IFITM3-deficient mice compared to control animals (**Fig 4C and 4D**). The presence of bead+ cells in the LN was dependent on DC migration as when beads were instilled into the airways of mice treated with pertussis toxin, a compound known to inhibit G-protein mediated migration of DC, no bead-carrying cells reached the lung-draining LN (**Fig 4E**).

A defect in the ability of IFITM3 KO DCs to traffic to the LN was also evident when we generated chimeric mice and restricted the IFITM3 deficiency to the bone marrow compartment. To do this IFITM3 deficient bone marrow was transplanted into wild type B6 mice, or B6 bone marrow was transplanted into IFITM3 KO mice. This resulted in chimeric mice that lacked IFITM3 expression on only the bone marrow derived cells (ie DCs) but not the parenchyma (IFITM3 KO→ B6) or mice that expressed IFITM3 on bone marrow derived cells but not the parenchyma (B6→ IFITM3 KO). Infection of these chimeric animals with influenza virus resulted in similar viral titres in the lung (**Fig 4F**). We instilled IAV and fluorescent beads intranasally into the chimeric mice and measured the number of DC bearing beads in the LN 48hrs later. We observed a reduction in the number of bead+ MHC II+ DC in the LN in the IFITM3 KO→B6 mice only (**Fig 4G**).

**Fig 5:**
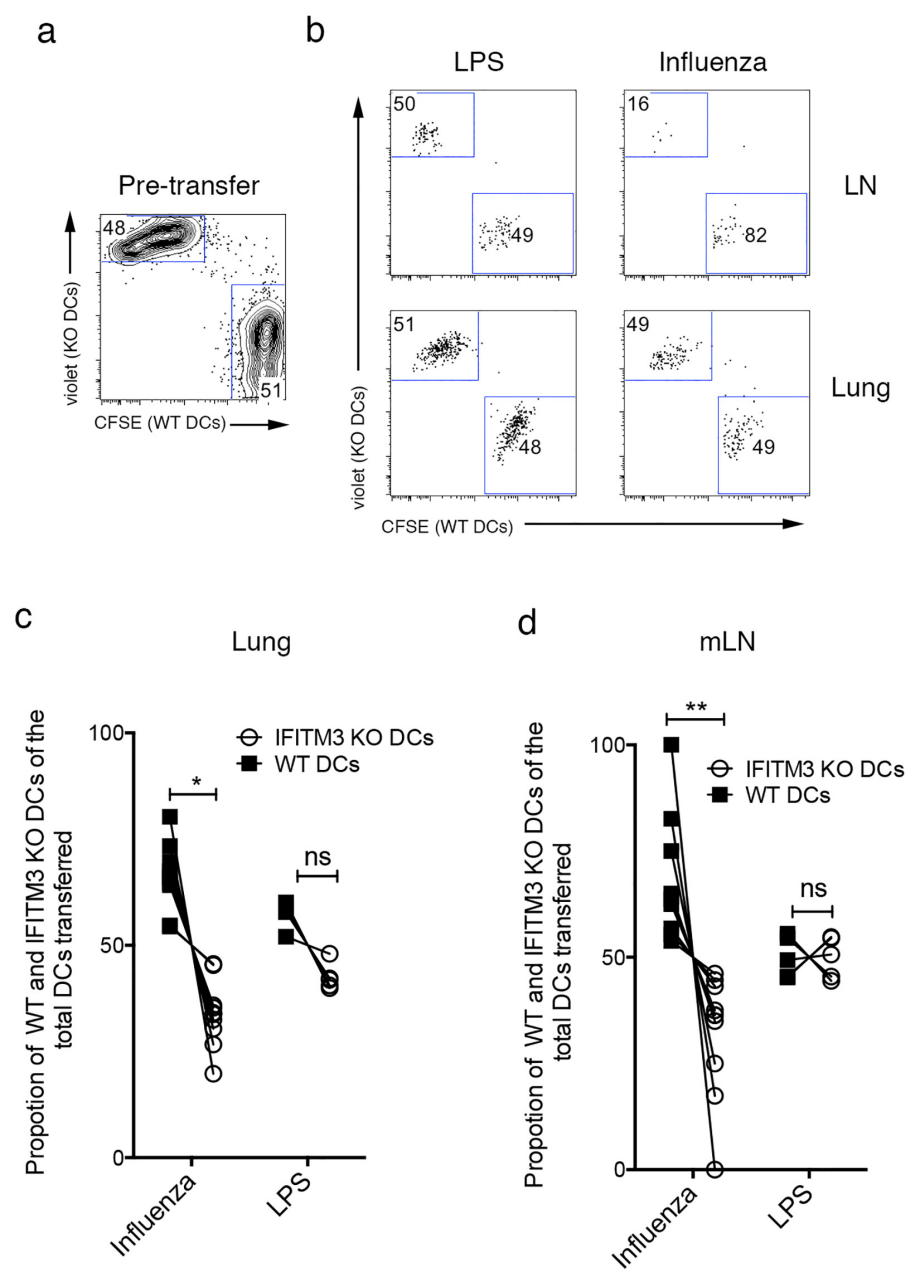
Impaired IFITM3 KO DC trafficking after intranasal instillation into the airway of influenza virus infected mice. B6 mice were infected with 10^4^ PFU of IAV (x31) and 24 hrs later received instranasally one million violet dye labeled IFITM3 KO and one million CFSE labeled WT DCs. A 1:1 ratio of DCs were also administered into control mice that were naïve and received LPS at the time of DCs immunization. Two days after DC administration the ratio of B6 and IFITM3 KO DCs in the lung and lung draining LN of the influenza virus infected and LPS treated mice was determined. (a) Representative flow cytometry profiles depicting the ratio of IFITM3 KO and WT DCs pre transfer and (b) 48hrs after transfer in the lung and LN. Profiles are gated on the adoptively transferred cells. The proportion of IFITM3 KO and WT DCs of the total DCs transferred in the (c) lung and (d) LN 48hrs after transfer into influenza infected or LPS treated mice. Lines join individual mice. (*n* = 5–10 mice per group, data pooled from 3 independent experiments, Student’s t-test **P <0.01).

Finally to compare IFITM3 KO and WT DCs migration to the LN in the presence of equal amounts of virus within the lung we adoptively transferred equal numbers of fluorescently labeled WT and IFITM3 KO monocyte derived DCs intranasally into the airways of B6 mice one day after intranasal IAV infection and tracked the migration of these cells to the lung draining LN 48 hrs later. We observed a significant decline in the proportion of IFITM3 deficient DCs in the lung and LN of the influenza virus infected mice (**Fig 5A–5D**) which was not evident when these DCs were transferred into the lungs of mice given an inert inflammatory stimulus (LPS).

The result of these experiments explain why the presentation of IAV antigen in lung-draining LN is defective in IFITM3-deficient mice, as arrival of DC capable of presenting viral antigen themselves, or of transferring the antigen to other LN resident DC, would be severely impaired in the mutant mice.

## Discussion

IFITM3 is a very potent antiviral molecule; it has previously been shown to contribute 50–80% of the resistance to IAV infection mediated by interferons [2]. Whether this activity is circumscribed to the lung epithelial cells where IAV infection is primarily localized, or also to other cell types, and in particular to cells of the immune system, has not been thoroughly investigated. We have previously shown that sustained expression of IFITM3 protects tissue resident memory CD8 T cells from death upon IAV infection of the lung mucosa and this activity is critical to protect mice from lethal IAV re-infections [5]. The present study extends the range of anti-viral functions of IFITM3 by demonstrating that its expression in respiratory DCs is essential for effective induction of a primary anti-IAV response in the lung-draining LN.

Generation of a robust cytotoxic T cell response is required for the clearance of IAV infection [14] [15]. This response depends on the ability of respiratory DC to transport viral antigen from the infected lung to the draining LN to either present the antigen themselves, or to transfer it to LN resident DC, which may then present the transferred antigen. The specific role played by each of the two respiratory DC subsets remains a contentious issue [8] [9]. The CD103+ DC has been shown to be resistant to IAV infection, and its survival is essential to ensure transport of viral antigen to the LN for CD8 T cell priming [16]. The antiviral state of CD103+ DC was dependent on type I interferon signaling [16]. We now show that induction of IFITM3 expression alone by type I IFN protects this DC type from viral infection, allowing its migration from the lung mucosa to the LN to exert its anti-viral function. The other DC subset contained in the lung mucosa, CD11b+ DC, were likewise protected from infection by IFITM3. Indeed, it is likely that all DC gain increased resistance to infection by IAV and other viruses by activating IFITM3 expression, a property that would be beneficial not only to preserve their antigen-presenting function but also to prevent viral dissemination. Being stationed throughout the body surfaces that often serve as the initial entrance point for infecting viruses, DC are amongst the first cells of the immune system to become infected. As one of the main activities of DC is to migrate from the site of infection to the closets draining LN, IFITM3-mediated protection of DC from virus infection may prevent dissemination of the pathogen to other cells within the LN [17]

In summary, our findings show that the expression of IFITM3 recruits anti-viral mechanisms in DC that play an essential role in protecting this cell type from virus infection, enabling initiation of anti-viral responses in the lung-draining lymph node and, potentially, preventing viral dissemination to lymphoid organs.

## Materials and Methods

### Mice and viruses

C57BL/6, B6.SJL-Ptprc^a^Pep3^b^/BoyJ (CD45.1), IFITM3 KO, OT-I.CD45.1, H2-Kbm1, OT-II x CD45.1, IRF3 KO, IRF7 KO and IRF7 x IRF3 KO mice were bred in-house and housed in specific pathogen-free conditions in the animal facility at the Department of Biochemistry and Molecular Biology, the University of Melbourne. IRF3, IRF7 and DKO were generously provided by Prof Taniguchi (University of Tokyo). All experiments were done in accordance with the Institutional Animal Care and Use Committee guidelines of the University of Melbourne. Mice were housed in IVC cages (5-mice/cage) and given food and water ad-lib. Mice were euthanized using CO_2_ administration. Mice were infected intranasally with 10^4^ PFU of x31-OVA [18] (encodes the OVA_257–264_ epitope within the neuraminidase stalk) generously provided by Dr. S. Turner, University of Melbourne, Melbourne, Australia.

### Adoptive transfer and T cell isolation

OT-I CD8 T cells and OT-II CD4 T cells were purified from the LN and spleen of OT-I, or OT-II, transgenic mice, respectively. Cells were purified after a depletion step using Abs against CD11b (M1/70), F4/80, Ter-119, Gr-1 (RB6), MHC class II (M5/114), and CD4 (GK 1.5) or CD8 (YTS 169.4), followed by incubation with anti-rat IgG-coupled magnetic beads (Dynal Biotech) following the manufacturer’s protocols. T cell preparations were 90–95% pure as determined by flow cytometry. 10^6^ purified CD8 OT-I.CD45.1 cells were labeled with 5 μM CFSE (Sigma-Aldrich) prior to intravenous injection into mice.

### Intranasal delivery of fluorescent beads

30 ul of 0.5 μm Fluorescent-conjugated plain microspheres (2.5% solids [wt/vol];Polysciences, Inc.), diluted 1:25 in PBS was instilled intranasally into the airways of mice. Pertusiss toxin and LPS was administered at a dose of 1ug intranasally.

### Mouse dendritic cell isolation from the lung and spleen

Total DCs were isolated from spleen tissue that was enzymatically digested with collagenase III and DNAse as previously described [19]. In brief, splenic cells were digested with Dnase I (Boehringer-Manheim, Mannhein, Germany) and collagenase III (Worthington Biochemicals, Freehold, NJ) and enriched for light-density cells by centrifugation in 1.077 g/cm^3^ Nycodenz (Nycomed Pharma, Oslo, Norway). Cells were isolated after a depletion step using antibodies against CD3 (KT3-1.1), Thy-1 (T24/31.7), Ter 119, Ly6G (RB68C5) and CD45R (RA36B2), followed by incubation with anti-rat IgG-coupled magnetic beads (Dynal, Oslo, Norway) following the manufacturer's protocol. Spleen DC were sorted into Sirpa+ (CD11c+Sirpa+CD24-CD11b+) and CD8+ (CD11c+Sirpa-CD24+CD103-) populations on an Aria III. Total DCs were isolated from lung tissue using the same protocol described for spleen DC isolation with the exclusion of the Nycodenz enrichment step. Lung DC were sorted into CD103+ (CD11c+MHCII+CD103+CD11b-F4/80-) and CD11b+ (CD11c+ MHCII+ CD11b+ CD103-F4/80-) populations on an Aria III.

### Flow Cytometry and cell sorting

Single cell suspensions were prepared from spleens by mechanical disruption. Mice were perfused prior to the harvest of the lung tissue which was enzymatically digested for 1 h at 37°C in 3 mL of collagenase type 3 (3 mg/mL in RPMI 1640 supplemented with 2% FCS). Cells were stained for 25 min on ice with the appropriate mixture of monoclonal antibodies and washed with PBS with 1% BSA. The conjugated monoclonal antibodies were obtained from BD Pharmingen or eBioscience.

### Ex vivo Ag presentation assay

B6 or IFITM3 KO mice were infected intranasally with Flu-OVA. The lung draining LN were removed, cut into small fragments, and digested in collagenase III (1 mg/ml; Worthington Biochemical) and DNase I (0.1%; Boehringer Mannheim). DC were purified and separated into subsets by cell sorting with a FACSAria III. Sorted DC subtypes (25,000) were co-cultured with 5 × 10^4^ CFSE-labeled OT-I cells and proliferation was assessed 60 h later by flow cytometry.

### In vitro Ag presentation assay

Spleen cells from H2-Kbm1 mice were incubated with 10 mg/ml OVA (Sigma-Adrich) in RPMI 1640 medium for 10 min at 37°C, and washed twice with RPMI 1640 medium supplemented with 2% FCS and then were gamma-irradiated (1500 rad). DC populations were added to 96-well Costar (Corning) plates at 10^4^ cells per well with different numbers of OVA-coated splenocytes at 37°C in RPMI 1640 medium supplemented with 10% FCS, 50 μM 2-ME, 2 mM L-glutamine, 100 U/ml penicillin, 100 μg/ml streptomycin, and 0.5uM CPG. CFSE-labeled OT-I or OT-II cells (5 × 10^4^) were added in each well. Proliferation was analyzed by flow cytometry after 60–65 h of culture. Each dilution was performed in duplicate.

### Real time PCR

Total RNA was extracted from cells following infection with influenza virus using an RNeasy kit (Qiagen, Venlo, Netherlands). Reverse transcription-PCR was performed using Superscript III First Strand Synthesis System for RT-PCR (Invitrogen) in combination with gene specific primers specific for influenza virus nucleoprotein following manufacturer's instructions. Influenza nucleoprotein RNA was amplified using the forward and reverse primers 5′-ACTCACATGATGATCTGG-3′ and 5′-CTGCATTGTCTCCGAAGA-3′, respectively. Real time PCR was performed using LightCycler 480 DNA SYBR Green I Master Mix (Roche Applied Science) following supplier’s recommendations. A standard curve was generated in each individual assay using serially diluted purified influenza viral cDNA.

For the measurement viral mRNA influenza virus infected cells were washed twice with PBS before RNA extraction by RNEasy Plus mini kit (Qiagen, Venlo, Netherlands). RNA purity and concentration was next verified by Nano-Drop 2000c (Thermo-Scientific, Waltham, USA) and samples of high purity 1.8< 260/280 ratio <2.1 were carried over for the further removal of trace genomic DNA contamination by in-solution DNAse I digestion (Sigma-Aldrich, St. Louis, USA). From each sample 40ng of template was used for Sensifast cDNA synthesis (Bioline, London, UK) and 4ng template used /well of each RT-PCR reaction.

RT-PCR was performed with Sensifast Lo-ROX SYBR Green (Bioline, London, UK) on a MX3005P (Stratagene, La Jolla, USA) using 300nM primers specific for GAPDH (5’ > 3’ CCAGGTTGTCTCCTGCGACTT, 3’ > 5’ CCTGTTGCTGTAGCCGTATTCA) and those previously described [20] for Influenza A virus M1/M2 transcripts (5’>3’ AAGACCAATCCTGTCACCTCTGA, 3’ > 5’ TCCTCGCTCACTGGGCA). Standard curves spanning relevant dynamic ranges were included in each experiment to quantitate the viral M1/M2 copy number and, as a measure of live infected cells, total input was normalised relative to GAPDH copy number. Final results are expressed as a ratio of normalised virus message levels of KO/WT.

For the measurement of IFITM3 expression, RNA was extracted using an RNeasy kit (Qiagen, Venlo, Netherlands), reverse transcription-PCR was performed using Superscript III First Strand Synthesis System for RT-PCR (Invitrogen) following manufacturer's instructions. Real time PCR was performed using RT^2^ qPCR Primer assays specific for murine *ifitm3* (Qiagen Inc. Valencia, CA) or primers specific for GAPDH (5’-GTC TCG CTC CTG GAA GAT GGT G-3’ and 5’- CAT TTG CAG TGG CAA AGT GGA G-3’) in combination with LightCycler 480 DNA SYBR Green I Master mix (Roche Applied Science) following supplier’s recommendations. Expression of ifitm3 was normalized to the housekeeping gene.

### Western blot

Total cell lysates were run on a 4–12% SDS/PAGE gel (Invitrogen) and transferred onto nitrocellulose membrane. Membranes were stained with antibodies against mouse IFITM3 (Abcam) and Actin (Sigma). Horseradish peroxidase (HRP)–conjugated anti-rabbit IgG antibody (Sigma) were used for protein detection.

### In vitro infection of DCs with influenza virus

DCs were cultured (10^5^ cells/well) in serum free media with influenza virus (moi 10) for 1 hour. Cells were then washed to remove free virus and cultured at 37°C in RPMI with 10% FCS for 12–24 hours. Infectability was measured by either detection of viral RNA or staining for intracellular levels of influenza nuclear protein (Abcam). Survival was measured by staining with a viability dye (eBioscience, fixable viability dye)

### Tracking in vitro generated bone marrow derived DCs

Bone marrow derived DCs were grown in the presence of GM-CSF and activated overnight with 1ug/ml of LPS as described previously [21]. DCs were labeled with membrane dye CFSE or cell trace violet prior to intranasal delivery.

### Immunofluorescence microscopy

LN, fixed in 100% Acetone and stained with the following combination of primary antibodies: anti-PNad, anti-MHCII (M5/114), anti-CD11c (N418)). Secondary antibodies were purchased from Molecular probes (anti-rabbit 594, anti-mouse 488, anti-goat 488). Images were acquired on a Zeiss LSM700 microscope with a 20x objective. Acquired images were processed using ImageJ software.

### Ethics Statement

All experiments were done according to Australian NHMRC guidelines contained within the Australian Code of Practice for the Care and Use of Animals for Scientific Purposes and under approvals given by The University of Melbourne Animal Ethics Committee (1312990)

## Supporting Information

S1 Fig IFITM3 KO DCs display normal expression of activation markers CD80 and CD86. B6 (blue histogram) or IFITM3 KO (red histogram) mice were infected via the intranasal route with 10^4^ PFU of influenza virus (x31) and 48 hrs later mice the lung draining LN was harvested and the level of expression of CD80 and CD86 on CD103+ and CD11b+ DCs (MHCII+ CD11c+) was measured by flow cytometry. Grey histograms represent isotype control staining.
(TIF)
