# Supplementary figures and images for: Respiratory DC Use IFITM3 to Avoid Direct Viral Infection and Safeguard Virus-Specific CD8+ T Cell Priming
Source: PLoS One. 2015 Nov 23;10(11):e0143539. doi: 10.1371/journal.pone.0143539 (PMC4657952; doi:10.1371/journal.pone.0143539)

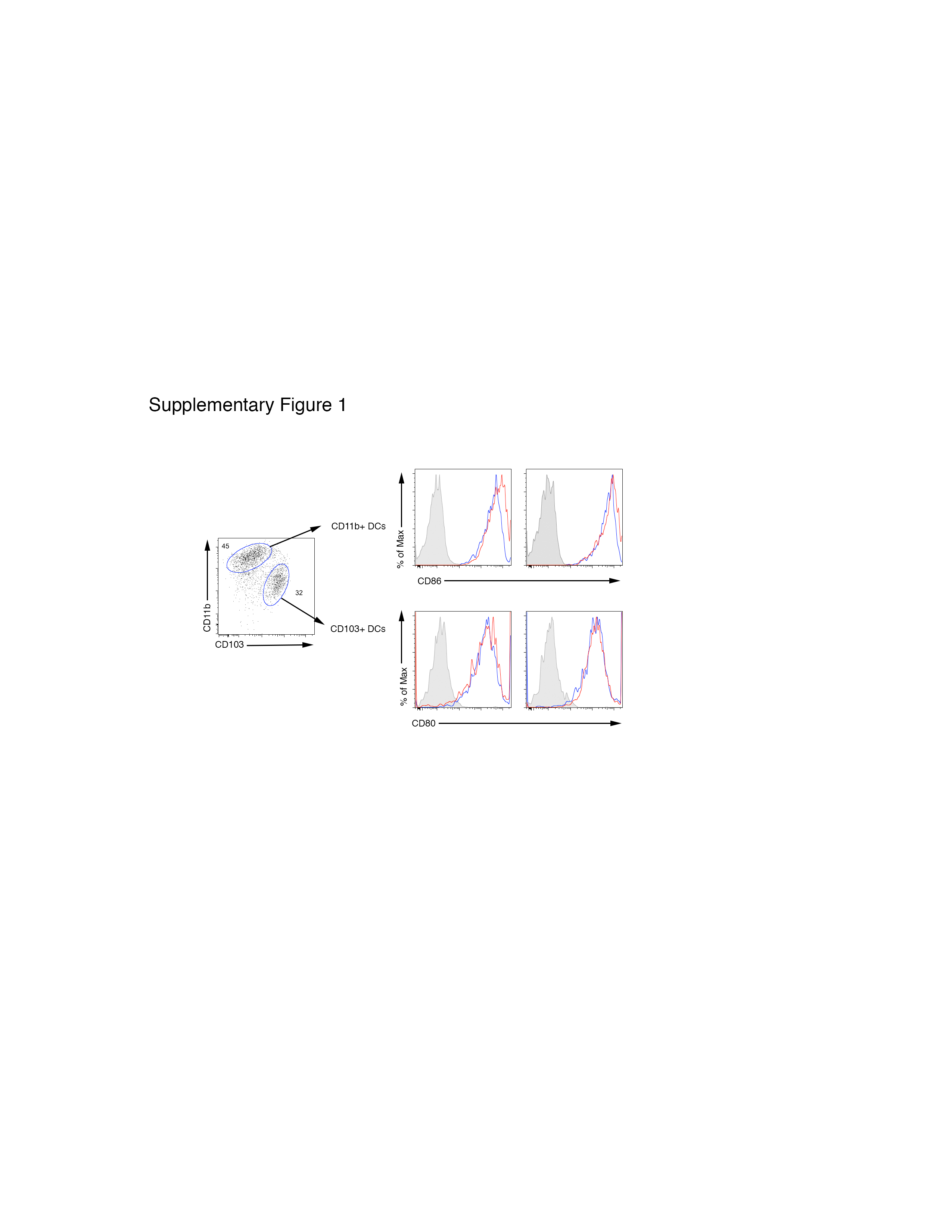

Supplement: S1 Fig — B6 (blue histogram) or IFITM3 KO (red histogram) mice were infected via the intranasal route with 104 PFU of influenza virus (x31) and 48 hrs later mice the lung draining LN was harvested and the level of expression of CD80 and CD86 on CD103+ and CD11b+ DCs (MHCII+ CD11c+) was measured by flow cytometry. Grey histograms represent isotype control staining. (TIF) [file pone.0143539.s001.tif]
